# 2424. Outcomes of vascular graft infections without graft removal treated via OPAT: A retrospective cohort study

**DOI:** 10.1093/ofid/ofad500.2043

**Published:** 2023-11-27

**Authors:** Oisin Hennigan, Laoise Geoghegan, Maura Reynolds, Elaine Morrison, Susan Clarke

**Affiliations:** St James's Hospital Dublin, Dublin 6W, Dublin, Ireland; St James's Hospital Dublin, Dublin 6W, Dublin, Ireland; St James's Hospital Dublin, Dublin 6W, Dublin, Ireland; St James's Hospital Dublin, Dublin 6W, Dublin, Ireland; St James's Hospital Dublin, Dublin 6W, Dublin, Ireland

## Abstract

**Background:**

Vascular graft infections (VGI) are a rare complication of endovascular aneurysm repair (EVAR) with significant associated morbidity and mortality. While surgical removal of infected material may be the preferred treatment, for patients who have inoperable VGIs conservative management with antibiotics may be the only option.

This study aims to investigate the outcomes including mortality, re-admissions, relapse within 12 months, microbiological data, bed days saved, and use of long term suppressive antibiotics in patients with VGI where the graft is retained.

**Methods:**

This single centre retrospective study evaluated all patients with a VGI between 2013-2021 who were treated via OPAT. Patient information was gathered from the local OPAT database and via the patients’ electronic patient records.

**Results:**

23/27 OPAT episodes were managed without graft removal. 52% were male. The median age was 70yrs (range 45-81). Median IV treatment of 6 weeks.

All patients received anti Staphylococcal cover while 14/23 episodes includes anti-MRSA agents.

6/23 episodes required re-admission over the course of OPAT treatment, of which 2 were OPAT related (1 DVT, 1 ADR) while 4 were disease related. 4/23 patients died within the first year of treatment all of which were secondary to their infected graft. In the remaining 19 patients there were no relapses by the 12 month follow up period.

13/16 patients who successfully completed their OPAT course were commenced on long term oral suppression.

32 bed days were saved per OPAT episode, a 77.0% reduction in overall hospital stay.

Type of infection

**Figure:**
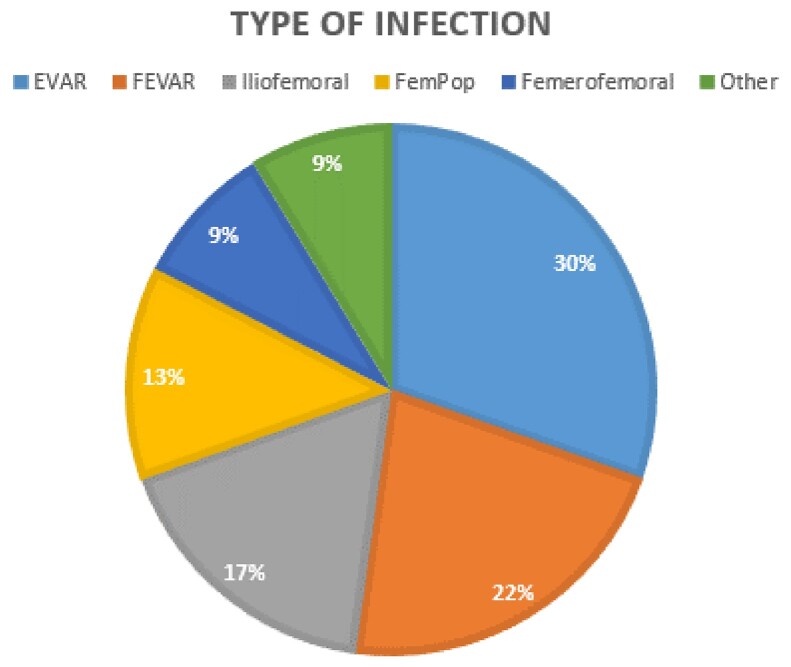

Pie chart breaking down the types of Vascular Graft Infections affecting the 23 patients included in this case series.

PO Suppressive Abx

**Figure:**
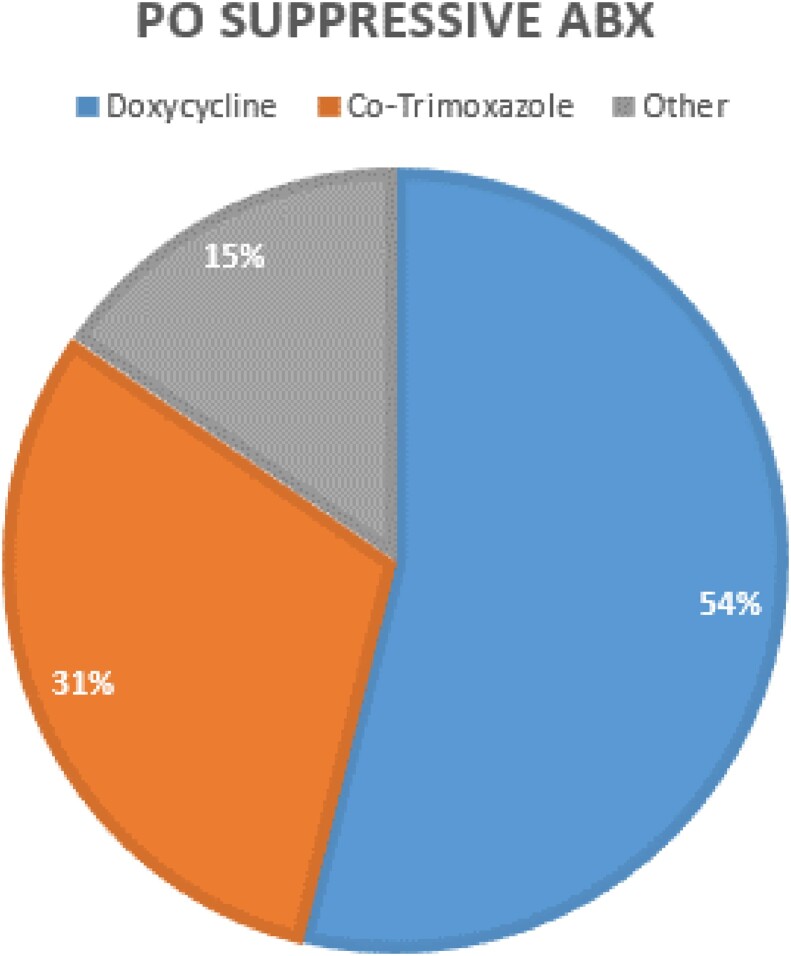

Pie chart displaying the PO suppressive antibiotics chosen for the 19 patients who completed OPAT successfully.

Culture results

**Figure:**
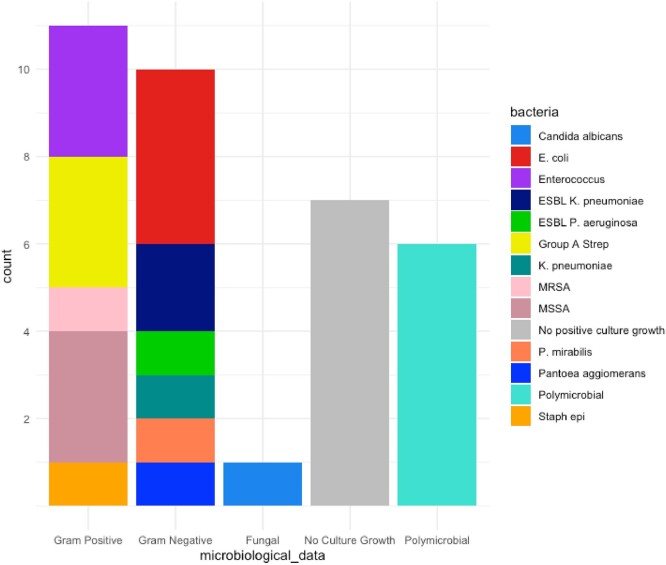

Bar chart displaying culture results. Sub categories include Gram-positive, Gram-negative, Fungal, Nil positive culture results and number of patients with polymicrobial culture results

**Conclusion:**

Limited data shows mortality associated with VGIs ranges from 25-55%. This study demonstrates successful outcomes in our cohort of patients treated conservatively without surgery but focussed antibiotic therapy long term. It was well tolerated in the OPAT setting after initial stabilisation in hospital and OPAT can be considered a safe option for VGI infections. The small study shows a 1-year survival rate of 82% comparing very favourably with other published data.

**Disclosures:**

**All Authors**: No reported disclosures

